# Research to develop a diagnostic ultrasound nomogram to predict benign or malignant lymph nodes in HIV-infected patients

**DOI:** 10.1186/s12879-023-08419-1

**Published:** 2023-07-10

**Authors:** Chen Huang, Xia Shi, Xin Ma, Jianjian Liu, Jingjing Huang, Li Deng, Ye Cao, Mingkun Zhao

**Affiliations:** 1 School of Medicine, Nantong University, Nantong, China; 2 Department of Vascular Surgery, Affiliated Hospital of Nantong University, Nantong, China; 3 Department of Ultrasonography, Shanghai Public Health Clinical Center, Fudan University, Shanghai, China; 4 Department of General Surgery, Shanghai Public Health Clinical Center, Fudan University, Shanghai, China

**Keywords:** Ultrasound, HIV, Lymph node, Diagnosis, Nomogram

## Abstract

**Background:**

This study aimed to establish an effective ultrasound diagnostic nomogram for benign or malignant lymph nodes in HIV-infected patients.

**Methods:**

The nomogram is based on a retrospective study of 131 HIV-infected patients who underwent ultrasound assess at the Shanghai Public Health Clinical Center from December 2017 to July 2022. The nomogram’s predictive accuracy and discriminative ability were determined by concordance index (C-index) and calibration curve analysis. A nomogram combining the lymph node US characteristics were generated based on the multivariate logistic regression results.

**Results:**

Predictors contained in the ultrasound diagnostic nomogram included age (OR 1.044 95%CI: 1.014–1.074 P = 0.004), number of enlarged lymph node regions (OR 5.445 95%CI: 1.139–26.029 P = 0.034), and color Doppler flow imaging (CDFI) grades (OR 9.614 95%CI: 1.889–48.930 P = 0.006). The model displayed good discrimination with a C (ROC) of 0.775 and good calibration.

**Conclusions:**

The proposed nomogram may result in more-accurate diagnostic predictions for benign or malignant lymph nodes in patients with HIV infection.

## Introduction

Whether benign or malignant tumors, lymphadenopathy is often the first manifestation of disease progression [1–6]. Lymphadenopathy is generally observed in infection or cancer due to the activation of the immune system and multiplied lymphocytes. Meanwhile, as a “pre-AIDS” syndrome, lymphadenopathy is also known as one of the first detected symptoms early in the AIDS epidemic [7–9]. However, previous studies have shown that the opportunistic infections, inflammatory conditions, and neoplastic processes will cause lymphadenopathy at any HIV infection stage [10]. Therefore, when a patient with HIV infection develops lymphadenopathy, it is necessary to identify the cause.

As one of the most common methods available, ultrasound has the characteristics of good discrimination of lymph nodes (LNs), non-radioactive, economical, and non-invasive [11]. The regular sonographic features of benign and malignant LNs on conventional ultrasound imaging include sizes, shapes, aspect ratios, margins, echogenicity/internal echoes, lymphatic hilum structures, matted LNs, lymph regions, necrosis, and intranodal vascular patterns/color Doppler flow imaging (CDFI) grades [5, 12–14].

Our study aimed to identify risk factors that may predict benign and malignant LNs in HIV-infected patients and build a nomogram by combining clinical and ultrasound features, which improves the accuracy of lymph node diagnosis by ultrasound in an objective manner.

## Materials and methods

Between December 2017 and July 2022 at the Shanghai Public Health Clinical Center, Fudan University, Shanghai, China, a total of 324 HIV-infected patients underwent ultrasound examination in our hospital and were diagnosed with lymphadenopathy. Inclusion criteria were as follows: (1) HIV-infected patients, (2) one or more regions with lymphadenectasis and the diagnosis were not yet clear, (3) pathological results of needle biopsy were available, and (4) ultrasonic and pathological images were available. The exclusion criteria were: (1) inadequate or indeterminate pathological results, (2) patients were under 18 years old, and (3) incomplete follow-up data. Of all 324 patients, 166 were not diagnosed by pathology, 2 were younger than 18 years old, and 25 had incomplete follow-up data. 131 HIV-infected patients who performed ultrasound tests and had pathological results of needle biopsy were included. This study was approved by the Research Ethics Committee of Shanghai Public Health Clinical Center, Fudan University, Shanghai, China.

### Ultrasound imaging characteristics

We used Mindray Resona 7 Color Doppler Ultrasound machine. All patients were examined in the supine position, and the examined sites were fully exposed. The frequency was set to 5.0–12.0 MHz, and the mode was color Doppler mode. The US imaging characteristics of each patient were retrospectively reviewed by two independent sonographers with more than ten years of experience in lymph node imaging; neither observer was aware of the clinical nor the pathological outcome. If the sonographers had different opinions, they met to determine their final decisions by consensus. Transverse and longitudinal images of the LNs to be diagnosed were obtained from each patient. We set the filter conditions to a minimum, with a blood flow velocity color scale range of 5.4 cm/s and a maximum scanning depth of 16 cm.

Lymph node vascularity was classified from grade 0 to 3 and evaluated by color Doppler flow imaging (CDFI) following the Adler criterion (1 within the tumor, 2 at the periphery of the tumor, 3 adjacent to the tumor, 4 random-when the tumor was not discretely seen on.

the color flow study) [15]. The aspect ratio was classified as ≤ 2 or > 2. The region of the lymph node was classified as single or multiple. If there was a suspicion of enlargement of the regional LNs for more than one, we defined it as multiple. The ill-defined margin was referred to as less than 50% margins of LN that could be visualized. Inhomogeneous internal echo was considered nonuniform within the LN. The matted lymph node was defined as the unclear capsule between LNs and the fused cortex of the LNs. Necrosis was considered a hypoechoic area within the LN without blood flow [16].

### Statistical analysis

Numerical variables were shown by mean ± SD, and categorized variables were summarized by absolute frequencies. Continuous variables were compared by the Student’s t-test, and categorized variables were compared by the χ2 test (or Fisher’s exact test as required) across two groups (benign and malignant). Univariate and multivariate logistic regression models were performed to estimate the capability of different variables in predicting the malignancy of LNs. Variables with P-value < 0.05 in the univariate analysis or variables identified by clinical experience were further included in the multivariate analysis. Results were considered significant at P < 0.05. Descriptive statistics and analyses were obtained using SPSS 26.0 (IBM corp., Armonk, USA).

Nomograms are statistical models that are ideally suited for individualized risk assessment. To provide the sonographers with a quantitative tool to diagnose the individual probability of malignant LNs, we built the diagnostic nomogram using the independent predictors selected by the multivariate logistic regression model to generate a combined indicator for estimating the likelihood of malignant LNs.

The nomogram was performed using the total points as a factor. Calibration curves were plotted to assess the calibration of the diagnostic nomogram, which was evaluated by plotting the predicted versus the actual probability for quintiles of the predicted probability of malignancy within a lymph node.

## Results

There were 131 patients total, including 80 (61.1%) benign and 51 (38.9%) malignant LNs. Among the patients, 112 were men, and 19 were women, with a mean age (± standard deviation) of patients of 43.82 ± 14.70 years (range: 18–76 years). Features of LNs in grayscale ultrasound were summarized in Table 1. There were significant differences in age, duration of HIV, number of enlarged lymph node regions, and CDFI types between benign and malignant LNs (all p < 0.05). However, there was no difference in sex, length of lymph node, aspect ratio, ill-defined margins, irregular shapes, inhomogeneous internal echo, unclearly lymphatic hilum structures, matted LNs, and necrosis between benign and malignant LNs (p = 0.103–0.971). Distributions of lymph node regions were listed in Table 2. The most common distribution regions were cervical (69/131 52.7%) and supraclavicular (41/131 31.3%). The pathological diagnoses of all lymph nodes were listed in Table 3.

**Table 1:** Clinical and US imaging characteristics of patients

Variables	Total (n = 131)	Benign (n = 80)	Malignant (n = 51)	p-value
Sex				0.185
male	112	71	41	
female	19	9	10	
Age (y)	43.82 ± 14.70	39.50 ± 12.70	50.59 ± 15.16	**< 0.001**
Duration of HIV infection (m)	33.60 ± 43.56	26.11 ± 31.08	45.36 ± 56.36	**0.029**
Number of enlarged lymph node regions				**0.011**
single	12	3	9	
multiple	119	77	42	
Length of lymph node (mm)	34.39 ± 17.95	32.18 ± 15.12	37.84 ± 21.36	0.103
Aspect ratio				0.893
< 2	89	54	35	
≥ 2	42	26	16	
Ill-defined margins				0.738
No	25	16	9	
Yes	106	64	42	
Irregular shape				0.392
No	39	26	13	
Yes	92	54	38	
Inhomogeneous internal echo				0.637
No	15	10	5	
Yes	116	70	46	
Unclearly lymphatic hilum structures				0.971
No	118	72	46	
Yes	13	8	5	
Matted lymph nodes				0.543
No	99	59	40	
Yes	32	21	11	
Necrosis				0.733
No	85	51	34	
Yes	46	29	17	
CDFI types				**< 0.001**
Grade 0–1	27	25	2	
Grade 2–3	104	55	49	

**Table 2:** Distributions of lymph node region

Lymph node region	Benign	Malignant
Single region		
One subdivision of submandibular	0	1
One subdivision of cervical	3	3
One subdivision of supraclavicular	0	2
One subdivision of axillary	0	2
One subdivision of inguinal	0	1
Multiple regions		
Bilateral parotid	1	0
Bilateral submandibular	1	0
Bilateral submandibular & bilateral inguinal	1	0
Bilateral cervical	17	4
Bilateral cervical & bilateral supraclavicular & bilateral axillary	1	0
Bilateral cervical & bilateral supraclavicular & bilateral axillary & abdomen & retroperitoneal	1	0
Bilateral cervical & bilateral axillary	2	1
Bilateral cervical & bilateral axillary & bilateral inguinal	2	1
Bilateral cervical & mediastinal & bilateral axillary	1	0
Bilateral cervical & abdomen	1	0
Bilateral cervical & abdomen & retroperitoneal	1	0
Bilateral cervical & retroperitoneal	0	1
Bilateral cervical & bilateral inguinal & abdomen & left-sided supraclavicular	0	1
Bilateral cervical & bilateral inguinal & retroperitoneal	0	1
Left-sided cervical	7	4
Left-sided cervical & bilateral supraclavicular	1	0
Left-sided cervical & splenic	0	1
Right-sided cervical	12	2
Right-sided cervical & bilateral supraclavicular	1	0
Bilateral supraclavicular	3	3
Bilateral supraclavicular & mediastinal	0	2
Bilateral supraclavicular & abdomen	1	0
Bilateral supraclavicular & retroperitoneal	1	0
Bilateral supraclavicular & bilateral inguinal	2	0
Left-sided supraclavicular	6	4
Left-sided supraclavicular & abdomen	0	2
Left-sided supraclavicular & abdomen & retroperitoneal	1	0
Left-sided supraclavicular & chest wall & axillary	2	0
Left-sided supraclavicular & mediastinal & abdomen & retroperitoneal	0	1
Left-sided supraclavicular & retroperitoneal	0	1
Left-sided supraclavicular & bilateral inguinal	0	1
Right-sided supraclavicular	2	2
Right-sided supratrochlear & chest wall	0	1
Bilateral axillary	0	1
Bilateral axillary & bilateral inguinal	1	0
Left-sided axillary	1	2
Left-sided axillary & abdomen	0	1
Right-sided axillary	0	2
Mediastinal	1	0
Abdomen	0	1
Abdomen & retroperitoneal	1	0
Abdomen & retroperitoneal & pelvic	1	0
Retroperitoneal	1	1
Right-sided inguinal	0	1
Bilateral inguinal	2	0
Bilateral inguinal & bilateral submental	1	0
Total	80	51

**Table 3:** Pathological diagnoses of all lymph nodes

Pathological Type	
Benign	Number
Tuberculosis	29
Nonmycobacterial tuberculosis	15
Marneffei infection	15
Lymphadenitis	9
Reactive hyperplasia of lymphoid follicles	4
Ebstein Barr Virus infection	2
Inflammation	2
Cytomegalovirus infection	1
IgG-associated sclerosing lymphadenopathy	1
S. aureus infection	1
Granulomatous inflammation	1
Total	80
Malignant	
Diffuse large B-cell lymphoma	10
Burkitt lymphoma	8
Metastatic adenocarcinoma	8
Hodgkin's lymphoma	6
Metastatic squamous cell carcinoma	4
Squamous cell carcinoma	3
High-grade B-cell lymphoma	2
Metastasis of papillary thyroid cancer	2
Peripheral T-cell lymphoma	2
Metastatic squamous cell carcinoma	2
B-cell lymphoma (undefined)	1
Warthin tumor/adenolymphoma	1
Follicular lymphoma	1
Metastasis of ductal carcinoma	1
Total	51

**Fig. 1:**
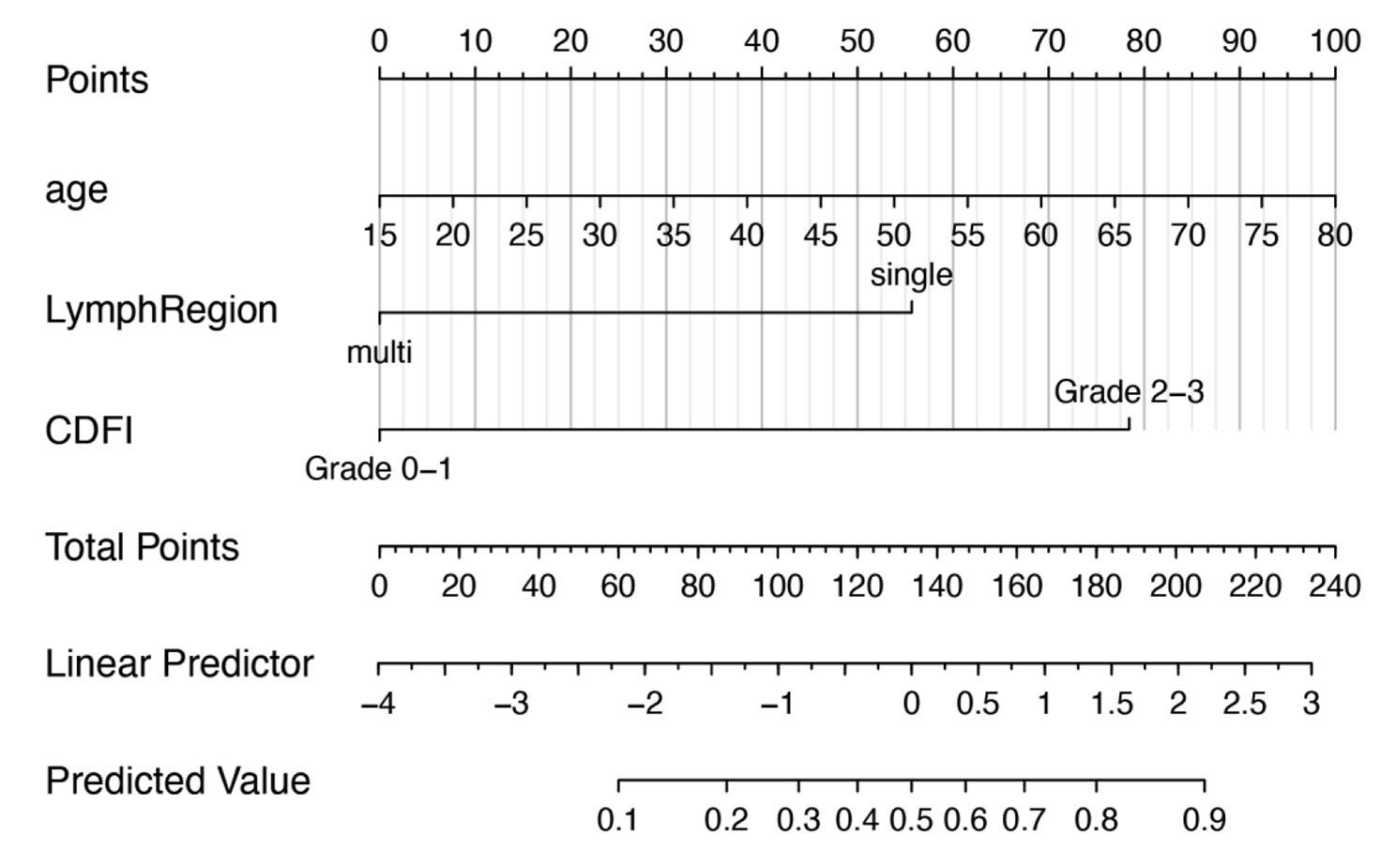
The nomogram was developed by the US reported LN status in the training cohort. CDFI, color Doppler flow imaging

Table 4 shows a univariate and multivariate analysis of important diagnostic factors for LNs. In the univariable analysis, the older age (OR 1.057 95%CI: 1.029–1.086 P < 0.001.

), longer duration of HIV infection (OR 1.011 95%CI: 1.002–1.020 P = 0.020), single enlarged lymph node region (OR 5.500 95%CI: 1.412–21.422 P = 0.014), and grade 2–3 of CDFI type (OR 11.136 95%CI: 2.508–49.455 P = 0.002) were associated with malignant lymph nodes. In multivariate analysis, the older age (OR 1.044 95%CI: 1.014–1.074 P = 0.004), single enlarged lymph node region (OR 5.445 95%CI: 1.139–26.029 P = 0.034), and grade 2–3 of CDFI type (OR 9.614 95%CI: 1.889–48.930 P = 0.006) were independent risk factors for the malignant lymph node. A model incorporating the above independent predictors was developed and presented as the nomogram (Fig. 1).

**Table 4:** Uni- and multivariate logistic regression analysis of risk factors for malignant lymph nodes

Variables	Univariate analysis		Multivariate analysis	
	Odds ratio (95%CI)	P-value	Odds ratio (95%CI)	P-value
Sex		0.190		
male	1 (reference)			
female	1.924 (0.723–5.122)			
Age (y)	1.057 (1.029–1.086)	< 0.001*	1.044 (1.014–1.074)	**0.004**
Duration of HIV infection (m)	1.011 (1.002–1.020)	0.020*	1.008 (0.998–1.018)	0.102
Number of enlarged lymph node regions		0.014*	5.445 (1.139–26.029)	**0.034**
single	5.500 (1.412–21.422)			
multiple	1 (reference)			
Length of lymph node (mm)	1.018 (0.997–1.039)	0.090		
Aspect ratio		0.893		
< 2	1 (reference)			
≥ 2	0.949 (0.447–2.018)			
Ill-defined margins		0.738		
No	1 (reference)			
Yes	0.857 (0.347–2.118)			
Irregular shape		0.393		
No	1 (reference)			
Yes	1.407 (0.642–3.084)			
Inhomogeneous internal echo		0.673		
No	1 (reference)			
Yes	1.314 (0.442–4.094)			
Unclearly lymphatic hilum structures		0.971		
No	1 (reference)			
Yes	0.978 (0.302–3.174)			
Matted lymph nodes		0.544		
No	1 (reference)			
Yes	0.773 (0.336–1.777)			
Necrosis		0.733		
No	1 (reference)			
Yes	0.879 (0.420–1.842)			
CDFI types		0.002*	9.614 (1.889–48.930)	**0.006**
Grade 0–1	1 (reference)			
Grade 2–3	11.136 (2.508–49.455)			

### Construction and validation of Nomogram

**Fig. 2:**
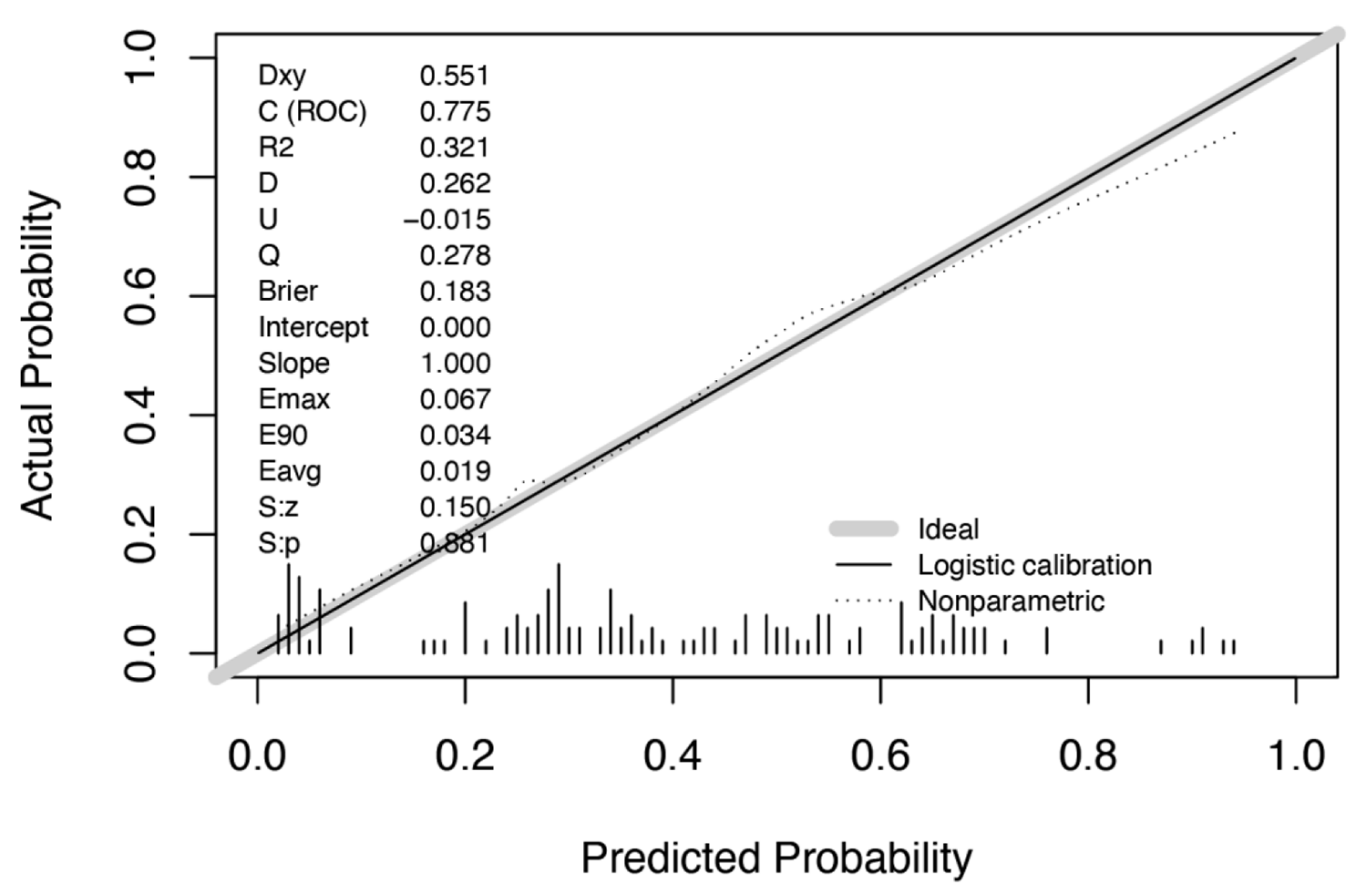
Calibration curve of the radiomics nomogram. C(ROC) for the prediction nomogram was 0.775 for the cohort. The calibration curve illustrates the calibration of the nomogram in terms of the agreement between the predicted risk of LNM and the observed outcomes of LNM. The 45° solid black line represents a perfect prediction, and the dotted gray line represents the predictive performance of the nomogram. The dotted gray line has a closer fit to the solid black line, which indicates better predictive accuracy of the nomogram

The age, number of enlarged lymph node regions, and CDFI types were selected to build the malignant LNs prediction nomogram (Fig. 1); the calibration curve of the malignant LNs nomogram for the prediction patients demonstrated good agreement in this cohort (Fig. 2). The C(ROC) for the prediction nomogram was 0.775 for the cohort, which suggested that the model has a good prediction. In the malignant LNs nomogram, apparent performance addressed the excellent prediction capability.

### Clinical use of the nomogram

**Fig. 3:**
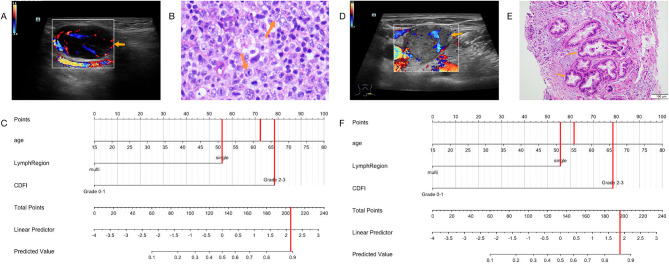
Example of the nomogram in use. **(A)** shows one of the multiple enlarged LNs (orange arrow) in the right axilla, and CDFI was a mixed type of hyperobstructed blood flow which was grade 3. Representative pathological image confirms diffuse large B-cell lymphoma. **(B)** The risk of malignancy calculated by nomogram was 89%. **(C)(D)** shows one of the multiple enlarged LNs (orange arrow) on the left suboclavicular bone and CDFI showed peripheral blood flow which was grade 2. Pathology confirms it was a metastatic adenocarcinoma. **(E)** The risk of malignancy calculated by nomogram was 86%. **(F)**

**Fig. 4:**
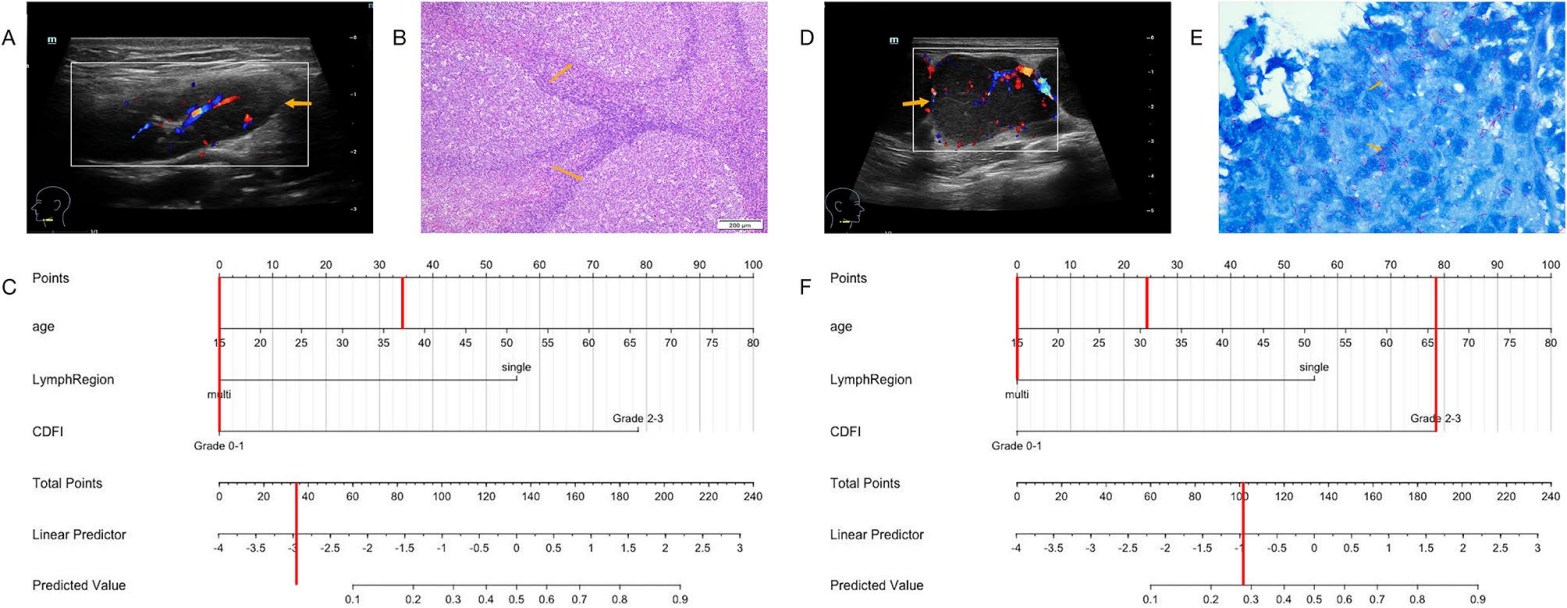
Example of the nomogram in use. **(A)** shows one of the multiple enlarged LNs (orange arrow) in the bilateral neck and CDFI showed portal blood flow with grade (1) Representative pathological image confirms reactive hyperplasia of lymphoid follicles. **(B)** The risk of malignancy calculated by nomogram was less than 10%. **(C)(D)** shows one of the multiple enlarged LNs (orange arrow) in bilateral neck and CDFI was mixed blood flow as grade (2) Representative pathological image confirms tuberculosis. **(E)** The risk of malignancy calculated by nomogram was 28%. **(F)**

We listed four examples of the use of the nomogram. The risk of malignancy in patient 1, who was 62 years old and had a multiple enlarged LNs in the right axilla, and CDFI was a mixed type of hyperobstructed blood flow which was grade 3 (Fig. 3A), could be calculated to be 89% (Fig. 3C). Pathology showed diffuse growth of numerous lymphocytes and diagnosed as diffuse large B-cell lymphoma (Fig. 3B). Patient 2, who was 55, had multiple enlarged LNs on the left subclavicular and CDFI showed peripheral blood flow which was grade 2 (Fig. 3D), has a risk of 86% (Fig. 3F). Pathology confirms it was a metastatic adenocarcinoma (Fig. 3E). Patient 3, who was 37 years old, had multiple enlarged LNs in the bilateral neck and CDFI showed portal blood flow with grade 1 (Fig. 4A). The malignant risk of this patient was less than 10% (Fig. 4B). The pathological diagnosis was a reactive hyperplasia of lymphoid follicles (Fig. 4C). Patient 4, who was 31, had multiple enlarged LNs in bilateral neck and CDFI was mixed blood flow as grade 2 (Fig. 4D), has a risk of 28% (Fig. 4F). Pathologist found inflammatory cell infiltration, granuloma formation, complete coagulative necrosis, acid-fast staining (+), and considered tuberculosis (Fig. 4E).

## Discussion

As far as we know, there are some pieces of research about using PET/CT for distinguishing malignant lymphoma from inflammatory lymphadenopathy in HIV-infected patients and endobronchial ultrasound in HIV-infected patients with mediastinal lymphadenopathy [17–19]. However, PET/CT is uneconomical and difficult to obtain. There are rare reports on using ultrasound, which is non-radioactive, economical, and non-invasive, to diagnose superficial LNs in patients with HIV-infected. Our study demonstrated that the older age, single enlarged lymph node region, and grade 2–3 CDFI types were independent US features differentiating malignant LNs from benign LNs in HIV-infected patients. Meanwhile, we developed and validated an ultrasound-based nomogram to improve the diagnosis of LNs with these 3 features of ultrasonography.

In the era of antiretroviral therapy (ART), the life expectancy of HIV-infected patients is significantly prolonged than before, and malignancy has become the leading cause of death among HIV-infected patients [20, 21]. Increased age was similarly observed in our study as an independent risk factor for the malignant lymph nodes in HIV-infected patients and was taken into consideration in the Nomogram analysis.

As the reservoir of HIV, LNs can be infected regionally within 3–6 days and systemically within 6–25 days after the initiation of the AIDS epidemic [22, 23]. Nevertheless, the sentinel LN is the first LN draining a primary tumor and harbors a high probability for metastatic seeding [24]. The sentinel lymph nodes are usually located in a specific area. In our study, the single enlarged lymph node region enlargement predicted a higher risk of malignancy, which we attribute to the fact that malignant lymph node drainage is more likely to be a specific region. In contrast, lymph node drainage in HIV-infected patients is more likely to be multiple regions.

CDFI technology can increase the specificity of ultrasound by providing real-time vascular assessment [25, 26]. In our study, CDFI was significantly different between benign and malignant lymph nodes, and the richer the lymph node blood supply, the higher probability of malignant LNs.

The subcapsular sinus of the lymph node is the first site in the tumor-draining lymph node contacted by tumor-draining material carried in afferent lymph [24]. Then the tumor-induced LN remodeling will happen, which means the increased lymphangiogenesis and angiogenesis induced by vascular endothelial growth factors (VEGFs), and dilation and de-differentiation of high endothelial venules (HEV) [27–29]. Then, the ultrasonography will show a peripheral type or mixed type of intranodal vascular patterns, which also known as grades 2–3 of CDFI types. However, in HIV or tuberculosis (TB), the changes of LN architecture are associated with constant immune activation and tissue inflammation, and it does not cause alteration of hemodynamics in the lymph nodes. On the contrary, due to the extensive deposition of collagen (fibrosis), the LN tissue gets damaged, and there may even be no blood flow, and the CDFI type is shown as grade 0–1 [27, 30].

There are still some limitations of our study. First, this is a retrospective study, and selection bias may occur. Second, the performance of our nomogram depends on the accuracy of the operator-reported imaging features, which may be subjectively biased. Finally, we need to perform external validation in a larger sample to obtain more objective conclusions. The model could also be improved by incorporating more valuable techniques, such as elastography and lymphangiography, which we intend to investigate in the future. However, in the current situation, it is necessary to obtain anatomopathological samples in all cases to avoid a missed diagnosis. This study greatly improves the ultrasonic diagnosis of benign and malignant lymph nodes in HIV-infected patients.

## Conclusion

This study identified the age, the number of enlarged lymph node regions, and CDFI grades as meaningful ultrasound diagnostic features and established the nomogram to give a more-accurate diagnostic prediction for benign or malignant lymph nodes in patients with HIV infection. It helps distinguish these diseases, and this easy-to-use scoring system can be conveniently applied to facilitate diagnosing HIV-infected patients with lymphadenopathy.

## Data Availability

The datasets used and analyzed during the current study are available from the corresponding author on reasonable request.
